# Private and Public Asylums
*On the Reform in Private Asylums. By H. Monro, M.B., Oxon. 1 vol., 8vo. On Public Asylums for the Insane of the Middle and Higher Classes. By T. Dickson, L.R.C.S.E. A pamphlet.


**Published:** 1852-07-01

**Authors:** 


### Art. VI.—PRIVATE AND PUBLIC ASYLUMS.*

We have before us two works relating to this subject, the one containing suggestions for the improvement of private asylums, the other advocating their entire abolition, and the substitution of public asylums in their place. The questions mooted in these works are of so much importance to the public, as well as to the interests of a large section of the profession, and well-being of the insane, that it was our intention to have entered at length into their consideration; but, upon glancing our critical eye a second time over the essays, we came to the conclusion that the arguments advanced by both authors in support of their respective views were so obviously fallacious, and the suggestions made so evidently impracticable, that we did not consider them entitled to more than a cursory notice. We make these observations with every feeling of respect for Dr. Monro, whom we believe to be an honourably-minded gentleman, *conscientiously* advocating the views developed in his essay. Although, however, he argues in favour of private asylums, and points out, in several portions of his essay, the great advantages which result from their establishment, nevertheless, the inevitable—the irresistible effect of his work will be, to shake public confidence in all private institutions for the treatment of the insane,

* On the Reform in Private Asylums. By H. Monro, M.B., Oxon. 1 vol., 8vo.
On Public Asylums for the Insane of the Middle and Higher Classes. By T. Dickson, L.R.C.S.E. A pamphlet.

and thus do an unconceivable degree of mischief to the cause of humanity. If Dr. Monro does not openly avow his hostility to private asylums, and unconditionally propose their destruction, he does what is tantamount to this, for he suggests such an amount of offensive supervision and control over the medical proprietors and superintendents of all these institutions, that would undoubtedly result in the retirement of every man of independent feeling and character from their management. We maintain, that no gentleman with any respect for himself or the honourable profession to which he is allied, would submit to the sacrifice which Dr. Monro calls upon him to make in order, as he declares, to remove from his shoulders all responsibility. The gist of Dr. H. Monro's views is contained in those portions of his work in which he strongly urges increased supervision, the appointment of an inferior grade of commissioners or inspectors, and monthly instead of quarterly visitation. The object of these suggestions is to remove from the medical superintendent, in difficult cases, all responsibility, and to transfer the *onus* entirely to the commissioners in lunacy. In the first place, if a medical man be deemed qualified to have a licence, and considered fitted to superintend a private institution for the treatment of the insane, surely he ought not to complain of the amount of his responsibility, or wish to place it upon the already too heavily laden backs of those deputed to officially inspect all public and private asylums. If this proposition were made to the board of commissioners, we could have no difficulty in divining what answer would be given. Imagine Dr. Monro, or any other enthusiast, advocating the necessity for a greater increase of supervision, and suggesting that the commissioners should take upon themselves the whole responsibility of discharging patients from private asylums, thus leaving the medical superintendent and proprietor entirely free and unfettered in his opinion, surely the reply to such a proposition would in substance be as follows: "We have granted a licence to a number of gentlemen in whom we repose confidence;" we have inquired into their character, and ascertained their attainments, and see no reason why they are not as fully qualified as ourselves to decide whether the patients under their immediate care ought or ought not to be retained in the asylum. They are, or are supposed to be, in daily communication with their patients; to be conversant with the character of each case; to have the confidence of the family of the invalid; and, therefore, in a position to form a right conclusion as to the necessity of prolonging the detention or discharging from treatment any given case that may have been the subject of inquiry. We think the medical superintendent of the establishment has no right to shirk the responsibility of his self-imposed position, and to thrust it upon the commissioners. They are upon all occasions ready to co-operate with the resident medical officer, and willing to aid him, to the best of their judgment, but it would be unjust and unreasonable to expect that the members of the medical profession are to place themselves at the head of private asylums for the treatment of insanity, to derive all the advantages, professional and pecuniary, resulting from such a connexion, to throw themselves into a comfortable arm-chair, consoling themselves with the reflection that they are free from all responsibility; and that, if any question of doubt or difficulty should arise, it is to be settled, and settled only, by the commissioners in lunacy!"

Dr. H. Monro would make the medical superintendent a mere *non-entity*, a *puppet* in the hands of the commissioners, an *automaton*, a *dummy*—in fact, a SHAM; for although *nominally* the proprietor and director of his *own* establishment, he is to be virtually powerless and paralyzed, divested of the liberty of thinking and acting in accordance with his own judgment! Whatever may be his *status* in the profession,—his degree of knowledge,—his elevation of character,—his amount of experience—these go for nothing in the estimation of Dr. H. Monro. He is said to hold a licence from the commissioners in lunacy—he is the resident superintendent of his own asylum, and yet is not to be considered qualified to decide as to the state of mind of any of his patients; and can take no step for their liberation, without the sanction of the commission in lunacy!

Dr. Monro says that his suggestion "is to make the commissioners and other public inspectors as responsible as possible for the conduct of private lunatic asylums—to remove all civil responsibilities from medical proprietors, as much as may be, and to leave them, what is quite sufficient, alone the medical care and the charge of carrying out the details of the economy of the house." Again, our author urges as a reason for thus wishing the medical officer to shuffle out of the responsibility of *taking care of his own patients* (for in reality it amounts to this), that the "responsibility (of the medical superintendent) is, on certain occasions, *intolerable*." Did it not occur to Dr. Monro that the said responsibility might prove as "intolerable" to the commissioners or sub-inspectors as to the medical superintendent himself?

The questions of difficulty which, according to Dr. Monro, occur, and for the solution of which he suggests that the commissioners should be appealed to, are, whether "a *partially insane person, who is legally sent to an asylum, should be retained or not?—should a convalescent patient, about whose continued residence there is much cause for doubt, be discharged or not?*" We have no hesitation in saying, if the medical superintendent be not competent *himself* to answer satisfactorily these simple interrogatories, and *honest* enough to give a disinterested opinion of the matter, the sooner he is deprived of his licence the better it will be for the interest of his patients and well-being of society. Dr. H. Monro really cannot expect that the large body of respectable physicians connected with private asylums would thus consent to write themselves down asses, and quietly and tamely submit to the degradation to which he would reduce them.

"It has been urged that the position in which I would place the officers of private asylums would be one of such subordination, and subject to such surveillance, as would render it most disagreeable to many honourable minds (most undoubtedly). I would ask such disputants whether of the twain is the most disagreeable to the feelings of a gentleman—to be acting under subjection to competent authority, and freed from liability to suspicions and charges, or to be independent, and in consequence subject to aspersions and suspicions of a most unwarrantable nature?"

Every man of proper feeling, conscious of rectitude of purpose, and feeling his *own* strength, would not hesitate in exclaiming, "Give me independence of action, and I will willingly brave the 'aspersions' and 'suspicions' to which it is said I expose myself." Again, Dr. Monro observes:—

"The changes which I suggest would drive the proprietor and medical officer somewhat into the condition of a government official—subject certainly to superior or general officers, but very far from incurring odium or degradation on that account. What would be lost in independence would, in my opinion, be more than compensated for by a position of greater credit."

Dr. Monro observes, "I have no doubt of the propriety of an asylum for a *complete maniac.*" "Complete maniac!" what does he mean? According to our experience, there is less hazard to the insane and to society from what Dr. Monro terms a "complete maniac," than from those cases of insanity where the morbid impressions are not so obviously and palpably manifested. "Complete maniac!"—why, every case of insanity is "complete" of its kind. The man who raves "from morn to night" incoherently is a "complete maniac;" he who designates himself, although sane upon all other points, to be "the Son of God," is a "complete maniac;" he who says that one leg is his own and the other Madame Vestris's, is completely insane: therefore we must confess our inability to see the distinction which Dr. Monro draws between the class he designates as "complete" and other forms of disturbed mind.

The only argument that may be urged by others in favour of Dr. Monro's views, is, that unhappily the present race of medical superintendents and proprietors of private asylums are a "*degraded, unprin-*

*cipled, dishonest, and rapacious* body of men." Such being, in the estimation of such low-mouthed slanderers, the lamentable fact, Dr. Monro, in his intense regard for the interests of the public, proposes that their functions should be restricted to the medical treatment of their patients; and that they should, so far as the discharge and detention of those entrusted to their care are concerned, be entirely at the mercy of the sub-inspectors who may be selected to relieve them of such anxious and responsible duties! Dr. Monro may repudiate this *interpretation* of his opinion; but, speaking in honest and intelligible English, we feel assured we have not exposed ourselves to the imputation of misconstruing his suggestions. Admitting Dr. Monro's first principle to be sound, what guarantee should we have that the additional commissioner, sub-commissioner, or inspector, would be more competent to decide the difficult, the extremely knotty questions which would be submitted to him, than the medical superintendent himself? We can imagine a body of sub-inspectors, with the best intentions, making the position of the medical proprietor so intensely "intolerable" by their ignorance, officiousness, and caprice, that a residence in one of our model prisons would be preferable to that of being associated with the management of a private asylum. We have no hesitation in saying that no gentlemanly and honourably-minded man could or would submit to the degrading surveillance and interference to which Dr. H. Monro's suggestions would subject them. If Dr. Monro's views were acted upon, no person who had any respect for his character would connect himself with a private asylum, and the disastrous effect would be, that these institutions would *necessarily fall into the hands of an inferior class of men, unfitted in every point of view to have the care and treatment of the insane!* Holding opinions totally at variance with those advanced by Dr. H. Monro, and advocated in his work, we maintain that it should be the object of the Government to do its utmost to *encourage medical men of high moral and professional character and experience to connect themselves with the management of asylums;* and, having secured the co-operation of men of humanity, judgment, and skill, to place in them the most unlimited confidence. The medical superintendents of asylums should be treated as honest and honourable men, incapable of dirty, mean, dastardly, and shuffling conduct; and, by reposing implicitly in their judgment and character for integrity, the greatest service would be rendered to the insane. If this course of action were adopted, in lieu of that proposed by Dr. Monro, a superior order of men would not hesitate to connect themselves with private institutions, and thus, by a process of "reflex action," our knowledge of the therapeutics and pathology of insanity would be materially advanced. There is, unhappily for the cause of science and the best interests of humanity, a class of writers and orators who take a delight in invariably referring to the *past*—of dwelling upon things as they *were*, not upon things as they *are*. These men have a morbid pleasure in always descanting upon the horrors of a "madhouse"—of dwelling upon the subject of "manacles," "strait waistcoats," "leg-locks," "muffs," "chains," and in endeavouring to work upon the weak nerves of a number of superannuated old gentlemen and hysterical young ladies, by painting in glowing and terrible imagery the frightful cruelties said to have been formerly practised by those who had the care of the insane. These gentlemen keep a stock of these appalling implements of cruelty on hand, and have them ready for exposure whenever they wish to excite a sensation, or keep alive a prejudice. We have witnessed some of these painful exhibitions of *human vanity*, and have been disposed to exclaim —*cui bono?* These frequent recurrences to the alleged barbarities of past epochs—this determination to look *only* upon the dark side of the picture—this unwillingness to acknowledge *that it is just possible* the orator may, in his indiscreet zeal, overstate the subject-matter of his indignant eloquence, must seriously injure the reputation of all engaged in the cure of the insane, and materially retard the progress of cerebral pathology. Such proceedings as these cannot certainly have a beneficial effect upon the public mind. How much more consonant to good taste, conducive to the public interest, and gratifying to the feeling of their hearers, if these pseudo-humanity-mongers—men always on the look-out for a grievance—were to submit to the inspection of those who listen to them a sketch of a well-conducted modern asylum, conducted by the skilful and kind physician, in accordance with the most modern discoveries in psychological science, suggesting at the same time the great importance of prompt treatment, early isolation, and *the certainty of cure in the premonitory stage of insanity*, if judicious *medical treatment be at once adopted*. We maintain that it is injurious to the public welfare and disastrous to the interests of the insane, to be thus dragging from the black records of the past, exaggerated accounts of the cruelties and neglect to which they were formerly unhappily subjected. Let these things be eternally buried with our recollection of the "thumb-screw," the "wooden boot," and other instruments of torture used in the dark ages.

Dr. Monro says, "I began my professional life with a strong prejudice against asylums." Indeed! We are astonished at this candid avowal; but we will do him the justice to say, that he admits that "this prejudice has gradually, not only faded away, but been supplanted by the opposite conclusion."*

* This gentleman's family have been connected with Bethlem Hospital for a period of *one hundred and forty* years.

Mr. Dickson takes up a bolder position than Dr. Monro, and proposes that private asylums should be altogether abolished, and public institutions be erected for the reception of the insane of the middle and higher classes.

We have carefully read Mr. Dickson's pamphlet, and we feel bound to express our regret that so much zeal and questionable English should have been wasted in pursuit of such a phantom. The idea of abolishing private asylums,—of substituting in their place *public* institutions, is so preposterously absurd—palpably impracticable,—that we are astonished that any reasonable man could force his mind to the belief that an idea of the kind could, for one moment, be entertained by any one acquainted with the real wants of the insane. Mr. Dickson might, with the same chance of success, endeavour to write down St. Paul's Cathedral, the Tower of London, or the Duke of York's column. If a proposition were submitted to the British Parliament for the abolition of private asylums, we do not believe that the suggestion would, for a single moment, be entertained. If a motion of the kind were made in parliament, the answer in all probability would be, "by what right do you propose to interfere with the freedom of the public will, or wish to deprive us of the privilege of placing our relatives in private asylums, and under the care of men in whose knowledge and practical sagacity we have unbounded confidence? You may succeed in your efforts to abolish all private institutions for the treatment of the insane, but no law can *compel* us to send our friends or relations to *public* asylums." We feel assured *such would be the feeling* of those whose duty it would be to entertain and discuss the proposition, should it ever be submitted to the consideration of the legislature. My son is seized with a paroxysm of insanity;—my daughter manifests signs of mental derangement;—my wife's state of mind requires her to be subjected to treatment away from the associations of home; I am, upon making inquiries, informed that the only asylum to which I can send my relation is a *public one;* that if confined there, it must be in the same building with pauper lunatics!" Such being the only alternative left for a delicate and sensitive mind, it will not be difficult to divine what the decision would be. There are thousands who would adopt any course rather than place their friends or relatives within the walls of a *public asylum,* however high may be its repute. Mr. Dickson will exclaim, "it was never my intention to propose that ladies and gentlemen should be confined in the same house with paupers;" but such *must be the case* if *public* be substituted for *private* asylum, for the author's suggestion that government should undertake to build asylums for the relatives of the aristocracy, is too quixotic to merit a moment's consideration. Surely Mr. Dickson could not seriously entertain such a proposition? What would our Chancellor of the Exchequer say to a motion of the kind? Should it ever unhappily be the case that only *one* class of institution existed for the reception and treatment of the insane, viz., public or COUNTY asylums, the certain,—the unavoidable,—the disastrous and deplorable consequence would be, that *a large body of insane persons would be kept at home undergoing no effective plan of treatment; or would be secreted in cottages or in lodgings, subjected to no medical or moral curative process, or degree of surveillance apart from that derived from an occasional visit of a medical attendant or relation!* Do those who argue in support of Mr. Dickson's proposition suppose that any legislative enactment could receive the support of the parliament or country, justifying the official authorities in saying to the relatives of the insane,—"You shall not keep your insane son or daughter at home, neither will we permit you to confine them in lodgings or in separate cottages, but we will *compel* you to send them to the wards of a public asylum." It would not be difficult to conceive the reception which would be given to such a gross attempt to outrage the liberty of the subject! Admitting that the wealthy and aristocratic portion of the community could be compelled to send their relations to a county asylum, would they be better situated there than in a private institution?* We really cannot perceive what would be gained by the change. In the treatment of insanity, that physician is the most successful who, to a knowledge of the general principles of his profession, devotes the greatest amount of attention to the study of individual cases; watching carefully the operation of remedies upon the varied phases of the disease. Is this practicable in our large county hospitals? In a recent number of the *Lancet*,† a correspondent asks the question, "how is it that for the last twelve months there has been only ONE resident medical officer at the *West Riding of Yorkshire* Asylum, where there are always upwards of 700 patients?" SEVEN HUNDRED INSANE PATIENTS *under the care of* ONE *medical man!!*

At Colney Hatch, the most recently constructed county asylum, there are only *two* resident medical men, having under their sole care more

* It is with pain that we feel ourselves compelled to refer to the gross abuses discovered by the commissioners in their recent visitation to Bethlehem Hospital, and which has led to a material alteration in the medical organization of this national hospital. We ask men connected with public asylums to look at home before they bring their great guns to bear upon private institutions, and assail in unwarrantable terms the medical proprietors of these asylums. The facts, said to be sworn to before the Commissioners in Lunacy, relative to the treatment of the patients in this hospital, are alleged to be anything but creditable to the officials connected with it.

† June 12, 1852.

than 1000 patients! We ask, is it possible—skilful, able, and active as the resident officers may be—to carry into effect, with such a medical staff, any actual curative process of medical or moral treatment, unless they adopt the suggestion of the physician in one of Molière's comedies, who, upon interrogating his hospital assistant as to the treatment he had pursued, was informed that he had on the preceding day bled the right ward and purged the left; then, replied the doctor, we will reverse matters to-day; please purge the right ward, and bleed the left! Considering the multifarious duties which devolve upon the medical officers of our county asylums—the number of patients they have daily to visit, the time occupied in recording the history of new admissions, in attending at the meetings of the board, in keeping the "case-book," the "daily journal," and in superintending the general management of the asylum, and servants—we maintain it to be *physically* impossible to do justice to the patients, or to pay that degree of attention to *individual* and curable cases so necessary in the treatment of the insane. The patients may be well fed, comfortably clothed, humanely treated; but we ask whether it is possible to devote that amount of time to *the physical and mental aspect of individual cases so necessary in any plan of treatment adopted for the purpose of arresting the progress of cerebral disease?* God forbid that we should cast any censure upon the able and distinguished men connected with our public asylums. The fault does not rest with them; it is of the *system* we complain. The county magistracy cannot reasonably expect any *one* or *two* to do the work which ought to be divided between *four* men; and that, too, with salaries much higher than the *present* rate of remuneration.

Mr. Dickson's pamphlet contains a very inexcusable, ungenerous, ignorant, and indiscriminate attack upon all proprietors of private asylums, whom he, with great want of proper feeling and with bad taste, designates as, "*money-making speculators*," and whom he places, with gentlemanly propriety, in the *same category with publicans!** 

As a specimen of Mr. Dickson's *literary* ability, we cite the subjoined passage:—

* "In this enlightened age (says the author) there are nearly 3000 persons confined in upwards of 150 establishments, called private 'licensed houses,' the proprietors of which have as great an objection to an empty house as a publican could have to a similar predicament."—p. 25. It would appear that the proprietors of some *public* asylums have as great an objection to "empty houses" as the proprietors of private establishments are said to have, otherwise why are they so eager to have the *private patients* transferred to *their own institutions?* We feel assured that Mr. Dickson's attack upon the respectable proprietors of private asylums will be repudiated by the great body of gentlemen associated with our valuable national county asylums.

"The sentiment of justice to fellow man comprised in the mandate, 'Do unto others as ye would they should do unto you'—and which has induced our general censure of a system which has witnessed, under its influence, who shall tell how much of wrong—cruel wrong?—wrong inflicted to conceal a wrong—wrong done in ignorance of right—wrong inflicted by brutality or indifference of keepers or their master—wrong inflicted by cupidity to gratify the cupidity of relatives, by blood or interest—wrong done in malice—wrong heaped by crime, upon evidence to still it down, till wrong had got its ends, and dug the grave, and dropped the earth upon the coffin lid of right;—the sentiment of justice nevertheless compels us to acknowledge," &c.

Perhaps our readers may be able to attach some idea to this torrent of eloquence! We confess that it is quite beyond the range of *our* comprehension.

Pope says, (very impudently, our fair readers will say,)

> "Woman and fool are two hard things to hit;—
> For true *no-meaning* puzzles more than wit."

We have felt much pain in being obliged to speak in such disparaging terms of the works of Dr. Monro and Mr. Dickson; believing, however, that if their suggestions were carried into effect, sad and fatal results would ensue to that class whose interests this journal was established especially to protect, we have considered it our duty thus to animadvert upon them. We hope when next we have the pleasure of meeting these gentlemen in print, it may be under happier auspices. We assure them it always affords us greater gratification to praise than to censure.

